# 317. Implementing lifestyle medicine innovations for preventing and reversing chronic diseases in HIV care at a Federally Qualified Healthcare Center

**DOI:** 10.1093/ofid/ofad500.388

**Published:** 2023-11-27

**Authors:** Rahul Anand, Shagun S Prabhu, Valerie R Ingram, Elena Pevar, Orett Brown

**Affiliations:** Quinnipiac University, Community Health Services, Hamden, Connecticut; Northeastern University, Unionville, Connecticut; Community Health Services, Hartford, CT, Connecticut; Community Health Services, Hartford, CT, Connecticut; Community Health Services, Hartford, Hartford, Connecticut

## Abstract

**Background:**

Patients living with HIV are a vulnerable population, with high burden of chronic diseases and challenging social determinants of health. Many receive care in Ryan White grant funded multidisciplinary clinics in the community providing an opportunity to reimagine care delivery using innovative practices like shared medical appointments (SMAs). We designed and implemented lifestyle medicine (LM) interventions using interprofessional teams including one of the first described SMA programs for patients with HIV. Our pilot describes an iterative patient-centered model transferable and scalable to other HIV clinics or FQHCs for delivering lifestyle medicine to patients with HIV and other chronic infections.

Learning objectives of oral presentation

**Figure:**
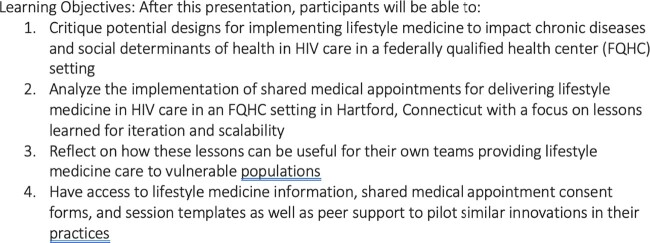

Learning objectives

Shared medical appointments recruitment flyer

**Figure:**
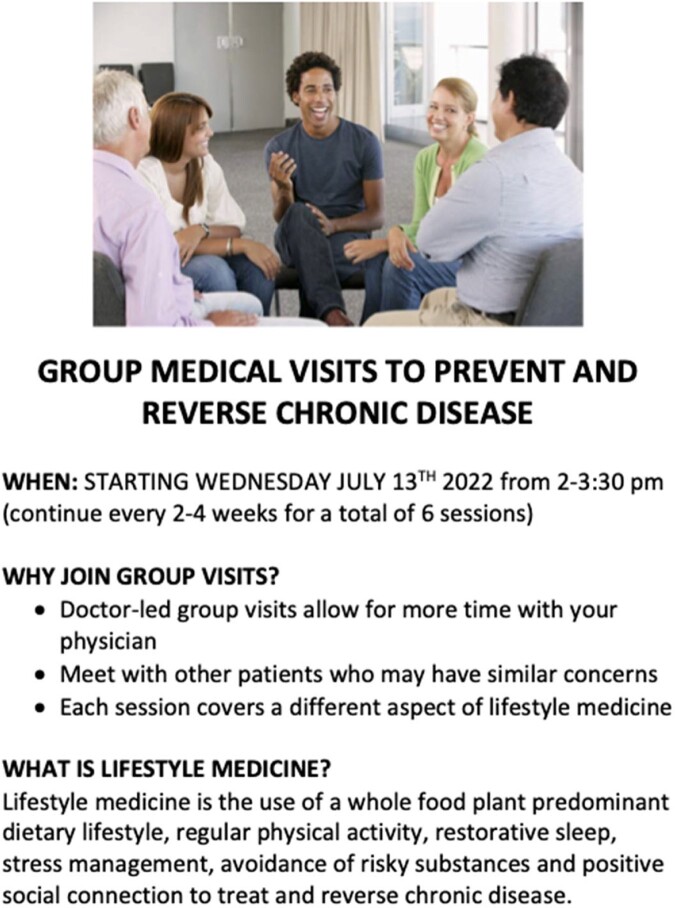

Recruitment flyer

**Methods:**

We piloted LM interventions both in individual patient care and via SMAs using interprofessional teams (physician, nutritionist, case managers). SMAs were 90 minutes long, designed to include 5-15 patients conducted monthly focusing on one pillar of LM at each event (nutrition,activity,sleep,stress,relationships,substance use). A whole health questionnaire was completed at the initial session, and American College of Lifestyle Medicine (ACLM) self-assessment on various pillars of lifestyle completed each session. Participants created a goal at the end of each session, and shared progress and key takeaways on return. Session guides were created for each session.

Lifestyle assessment page 1 of 2

**Figure:**
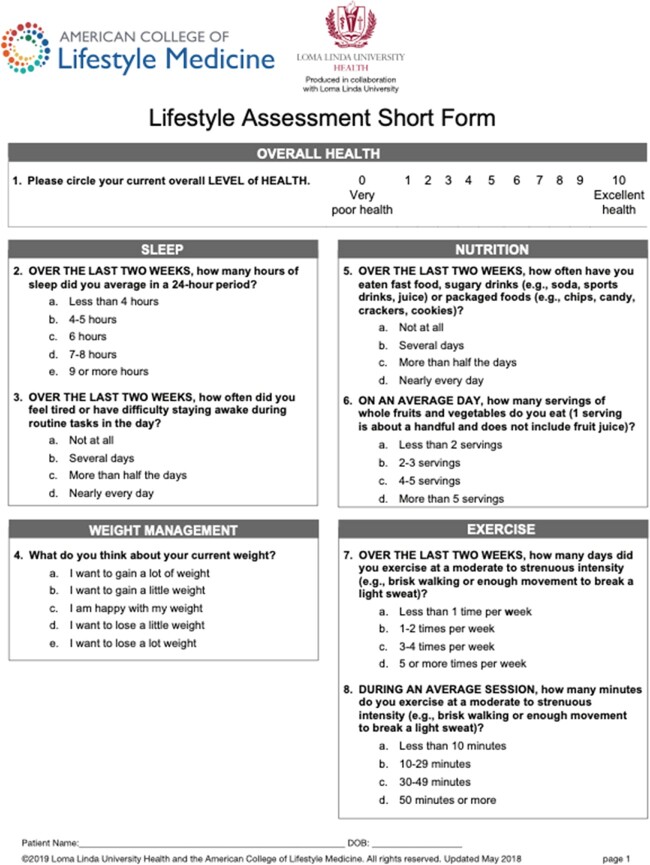

Lifestyle assessment page 2 of 2

**Figure:**
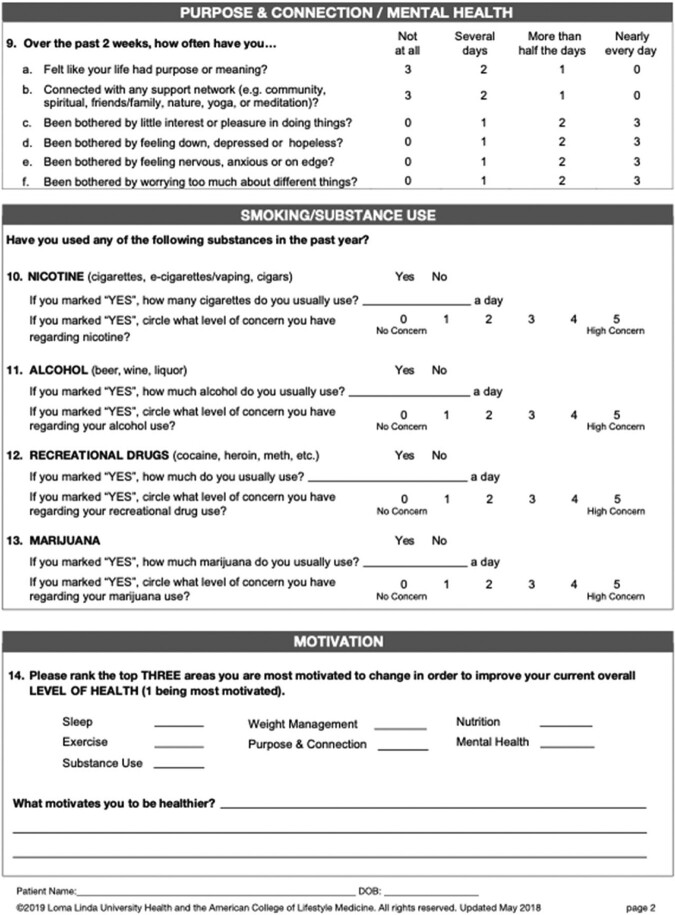

**Results:**

Serial assessments at SMAs demonstrated tangible and sustained changes in various pillars of LM. Patient feedback indicated high patient and provider satisfaction; patients found group visits “grounding” in their lives. Disclosing HIV status to other patients was not considered a barrier by any attendees. Other key lessons learned include the benefit of social support for patients, ease of conducting repeated assessments of the six pillars of LM using ACLM form, benefit of creating session detailed guides for future iterations and scalability, and the importance of signing up an adequate number of patients for financial sustainability.

Lifestyle medicine shared medical appointments outcomes graphic

**Figure:**
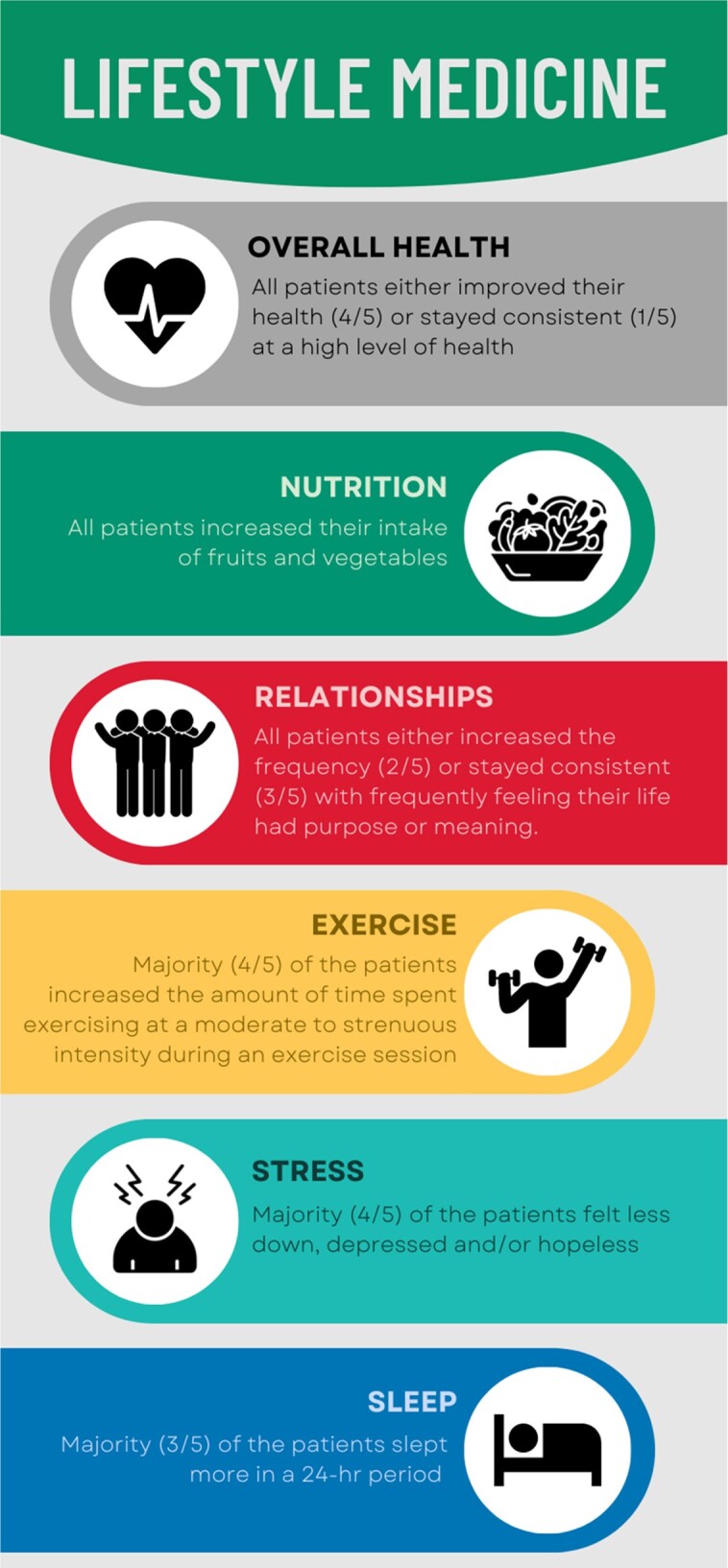

SMA Outcomes

Pre vs post program assessment data details for consenting patients

**Figure:**
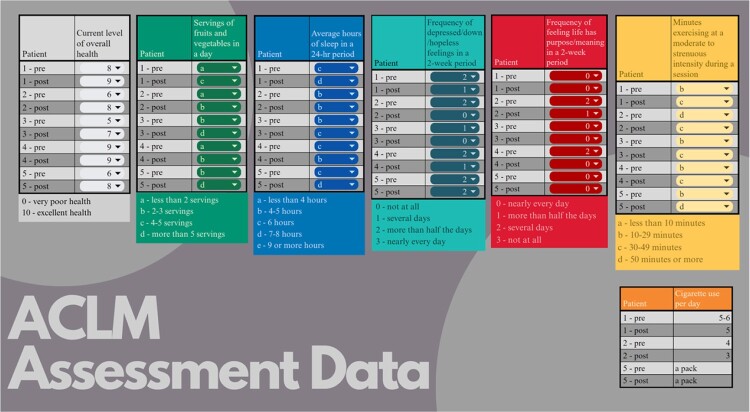

Pre vs Post program assessment data

**Conclusion:**

Our pilot provides an iterative patient-centered model of SMAs easily transferable and scalable to other practices for delivering lifestyle medicine care to impact chronic diseases affecting patients with HIV and other vulnerable populations.

**Disclosures:**

**Rahul Anand, MD MBA MSCI**, Paratek pharmaceuticals: Stocks/Bonds

